# Surgical pulmonary embolectomy on VA-ECMO

**DOI:** 10.1016/j.rmcr.2021.101551

**Published:** 2021-11-10

**Authors:** Rachel Steinhorn, Adam A. Dalia, Edward A. Bittner, Marvin G. Chang

**Affiliations:** Massachusetts General Hospital, Department of Anesthesia, Critical Care and Pain Medicine, United States

**Keywords:** Case report, Pulmonary embolectomy, Pulmonary embolism, VA-ECMO, Cardiopulmonary bypass, Extracorporeal membrane oxygenation, ACT, activated clotting time, ASD, atrial septal defect, CI, cardiac index, CPB, cardiopulmonary bypass, CT, computed tomography, EEG, electroencephalogram, ICU, intensive care unit, LPA, left pulmonary artery, MPA, main pulmonary artery, MPAP, mean pulmonary artery pressure, MRI, magnetic resonance imaging, PA, pulmonary artery, PE, pulmonary embolism, PERT, pulmonary embolism response team, PTT, partial thromboplastin time, PFO, patent foramen ovale, RPA, right pulmonary artery, SDH, subdural hemorrhage, TEE, transesophageal echocardiography, TPA, tissue plasminogen activator, VA-ECMO, venoarterial extracorporeal membrane oxygenation

## Abstract

Surgical pulmonary embolectomy is a procedure that is often used to rescue patients with massive pulmonary embolism (PE) and circulatory collapse that have failed or may not be ideal candidates for other systemic and endovascular treatment modalities. This procedure typically involves a sternotomy and the use of cardiopulmonary bypass (CPB), which requires full systemic anticoagulation. Here, we report the case of a surgical pulmonary embolectomy performed on venoarterial extracorporeal membrane oxygenation (VA-ECMO) rather than CPB to minimize systemic anticoagulation. The patient had suffered a cardiac arrest due to a saddle PE and required VA-ECMO which was complicated by a concomitant intracranial hemorrhage. The patient tolerated the surgical pulmonary embolectomy performed on VA-ECMO without procedure-related complications, and the ECMO support did not substantially complicate the technical performance of the procedure. In contrast to surgical pulmonary embolectomy performed on CPB, greater attention must be paid to volume status when performing the procedure on VA-ECMO since there is no blood reservoir. This case suggests cardiopulmonary support on ECMO as a viable strategy for surgical embolectomy in patients with unstable PEs in whom thrombolysis or full systemic anticoagulation are contraindicated.

## 1 Introduction

Surgical pulmonary embolectomy is a procedure that is often used to rescue patients with massive pulmonary embolism (PE) and circulatory collapse that have failed or may not be ideal candidates for other systemic and endovascular treatment modalities. This procedure typically involves a sternotomy and the use of cardiopulmonary bypass (CPB) which requires full systemic anticoagulation. Here, we report the case of a surgical pulmonary embolectomy successfully performed on venoarterial extracorporeal membrane oxygenation (VA-ECMO) rather than CPB to minimize systemic anticoagulation for a patient with a saddle PE and cardiac collapse requiring VA-ECMO support and unable to receive full systemic anticoagulation due to a concomitant intracranial hemorrhage.

## 2 Case report

A 48-year-old man with a history of a traumatic fall and subdural hematoma requiring evacuation 3 months prior presented to an outside hospital after an unwitnessed fall. At the time of presentation, he reportedly suffered three short (2 minute) cardiac arrests with return of spontaneous circulation, after which he received tissue plasminogen activator (TPA) for a presumed massive PE. On subsequent computed tomography (CT) imaging, the presence of a saddle PE was confirmed along with an acute multifocal intracranial hemorrhage, which included a 4mm subdural hemorrhage (SDH) without midline shift. TPA was discontinued and aminocaproic acid was administered. He was transferred to our tertiary care hospital with escalating vasopressor requirements (norepinephrine 40mcg/min and vasopressin 0.04u/min), and after consultation with our multidisciplinary PE response team (PERT), the decision was made to place the patient on VA-ECMO as a bridge to facilitate surgical pulmonary embolectomy. The right common femoral vein was cannulated with a 23/28 French venous cannula, and the left common femoral artery was cannulated with a 19 French arterial cannula, which included a 7 French distal perfuser.

Alternatives to surgical pulmonary embolectomy were considered. Catheter directed thrombolysis was determined not to be appropriate given that the patient had already received nearly systemic lytic dosing with continued hemodynamic instability and thrombus burden. Mechanical thrombectomy was considered, but it was felt that a surgical thrombectomy would be faster and more efficacious if he was a surgical candidate.

In consultation with the neurosurgery service, serial neurologic exams and head CTs were performed over the first 12 hours of admission which showed no interval worsening of the intracranial hemorrhage while receiving a heparin infusion with partial thromboplastin time (PTT) goal of 50–70 seconds. On the second day of admission, head CT demonstrated interval progression of the intracranial hemorrhage with an increasing midline shift of 4mm. Brainstem reflexes remained intact, although electroencephalogram (EEG) showed burst suppression and he had no response to voice or painful stimuli. On optimal medical therapy with VA-ECMO flow at 4.8LPM, in addition to inotropy with epinephrine and milrinone infusions, transesophageal echocardiography (TEE) showed a severely dilated, hypokinetic right ventricle, a decompressed left ventricle with normal function, the absence of a patent foramen ovale (PFO), and a large saddle embolus. The decision was made to proceed with a surgical pulmonary embolectomy to expedite weaning from ECMO and avoid continued systemic anticoagulation in the setting of expanding intracranial hemorrhage.

Given the evidence of a worsening intracranial hemorrhage, a decision was made to perform the procedure on ECMO rather than on CPB in order to minimize the need for systemic anticoagulation. A peripheral rapid infusion catheter (RIC) was placed prior to the procedure and attached to a Belmont rapid infuser, adequate blood products were available, and a cell saver system setup to facilitate volume resuscitation. The cardiac surgeon performed a median sternotomy, the main pulmonary artery (PA) (MPA) was opened, and a Foley catheter was passed proximally in an attempt to occlude the right ventricular outflow tract. Due to the high rate of forward flow through the MPA, the surgeon could not adequately confirm safe placement of the Foley within the right ventricular outflow tract (RVOT). Due to concern for catastrophic consequences if the catheter was passed and the balloon inflated distally rather than proximally, a cross clamp was placed across the MPA to control bleeding. The saddle PE was then extracted from the MPA. The left pleural space was opened, and the surgeon attempted to manually extrude any remaining thrombus from the left PA (LPA) into the MPA. An endotracheal suction was then passed from the MPA into the LPA, with no residual thrombus noted. The same maneuver was repeated for the right PA (RPA), again with no residual thrombus noted. Euvolemia was maintained during the procedure with volume resuscitation of 1200 cc of cell saver blood and 5 units of packed red blood cells delivered via the Belmont rapid infusing device.

**Fig. 1:**
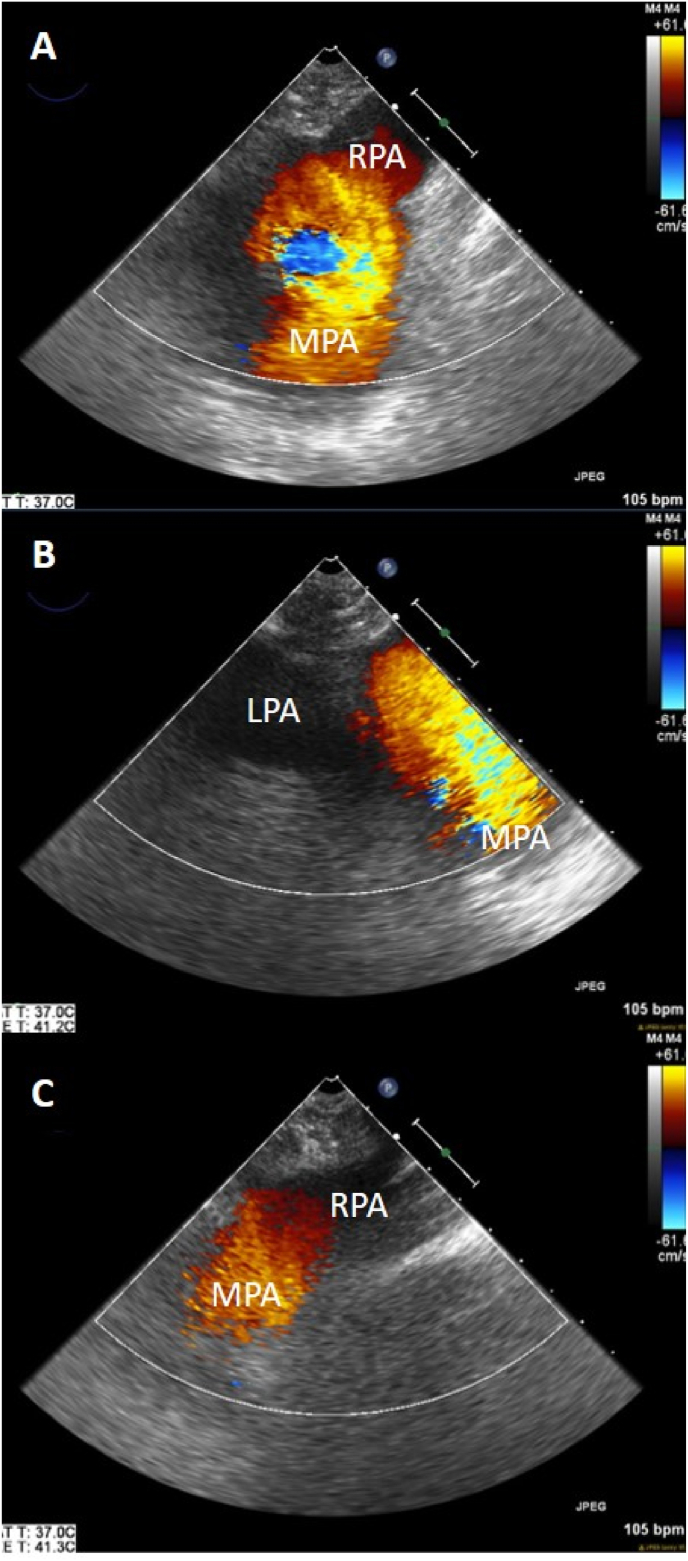
Intraoperative transesophageal echocardiogram images with color flow doppler of the main pulmonary artery (MPA), left pulmonary artery (LPA) and right pulmonary artery (RPA) prior to surgical pulmonary embolectomy. The images reveal significant flow acceleration across a dilated MPA (Panel A) and minimal to no antegrade flow into the LPA (panel B) and RPA (panel C). (For interpretation of the references to color in this figure legend, the reader is referred to the Web version of this article.)

**Fig. 2:**
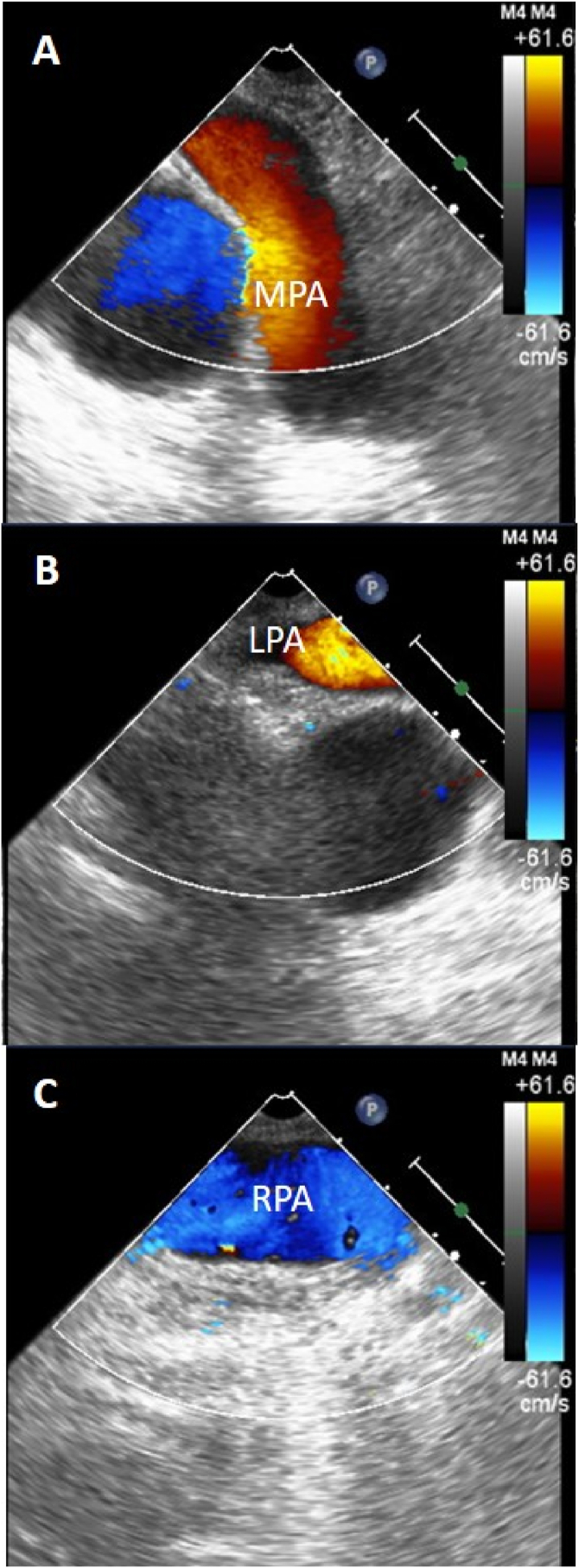
Intraoperative transesophageal echocardiogram images with color flow doppler showing the main pulmonary artery (MPA), left pulmonary artery (LPA) and right pulmonary artery (RPA) after surgical pulmonary embolectomy. The images reveal reduced flow acceleration across the MPA (Panel A) and significantly improved flow in the LPA (Panel B) and RPA (Panel C). (For interpretation of the references to color in this figure legend, the reader is referred to the Web version of this article.)

**Fig. 3:**
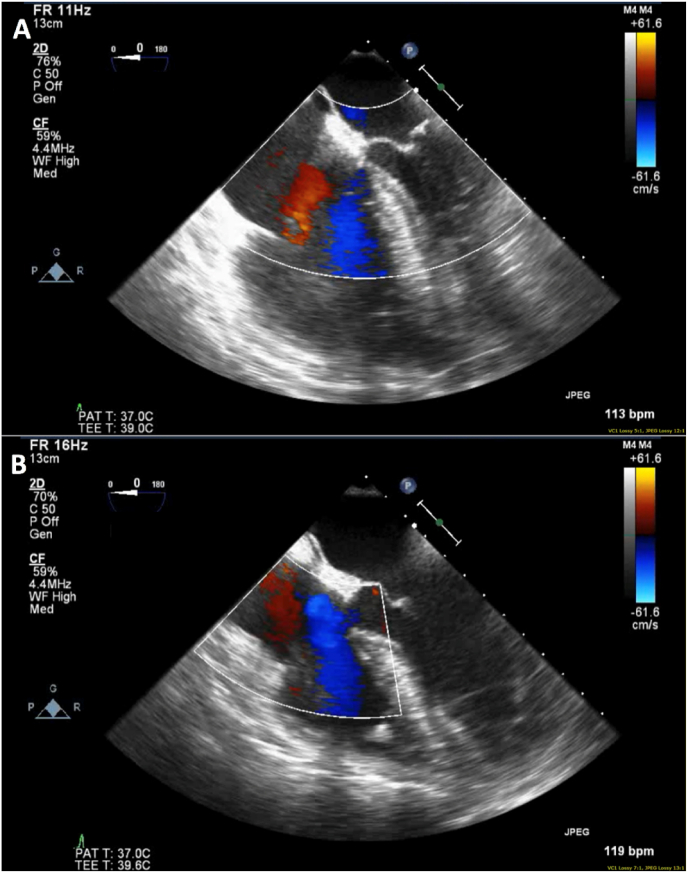
Intraoperative transesophageal echocardiogram images showing a significantly dilated right ventricle (Panel A) that normalized (Panel B) after surgical embolectomy.

Fig. 1 shows TEE images of the MPA, LPA, and RPA prior to embolectomy, which shows flow acceleration across a dilated MPA and minimal to no antegrade blood flow into the LPA and RPA. Fig. 2 demonstrates a reduction in flow acceleration across a markedly less dilated MPA and a significantly increased antegrade blood flow across the LPA and RPA. Fig. 3 shows the immediate reduction in right ventricular dilation after surgical embolectomy, with no change in the inotrope dose administered.

The patient returned from the operating room to the intensive care unit (ICU) receiving on milrinone and clevidipine, and he was decannulated uneventfully from VA-ECMO later in the day. After being weaned off all vasoactive support and pulmonary vasodilators, he had a cardiac index (CI) of 4.3 L/min/m^2^ and a mean pulmonary artery pressure (MPAP) of 21 mmHg. Unfortunately, the patient's neurologic exam failed to improve off sedation, and in conjunction with a magnetic resonance imaging (MRI) demonstrating diffuse anoxic injury, the neurology and neurosurgical consulting services agreed that there was limited potential for any meaningful recovery. The patient's family did not believe further intensive care support was within his goals of care, and he was transitioned to comfort measure only and terminally extubated on postoperative day 5 with family at the bedside.

## 3 Discussion

ECMO has previously been utilized for hemodynamic support to facilitate mechanical thrombectomy and even catheter-directed thrombolysis for both PE and ischemic strokes [1,2], but ECMO has not previously been reported to facilitate surgical embolectomy. Surgical embolectomy is typically performed on CPB with bicaval cannulation. CPB requires a higher level of systemic anticoagulation compared to VA-ECMO maintenance, with a typical target activated clotting time (ACT) of >400 for CPB and 180–200 for VA-ECMO according to institutional protocol. This is primarily due to the presence of a venous reservoir in a CPB circuit where there is significant stasis of blood [3]. In this patient with evidence of worsening intracranial bleeding, further thrombolytic therapy was clearly contraindicated, and the increased level of anticoagulation necessitated by transitioning to CPB would have risked further exacerbation of the intracranial hemorrhage.

The presence of a reservoir facilitates adequate volume resuscitation on CPB in cases complicated by significant hemorrhage. Avoiding hypovolemia while on ECMO is more challenging due to the lack of a reservoir; it therefore requires close attention to direct and indirect measures of blood loss, including monitoring the quantity of blood captured in surgical suction and sponges, hemodynamic parameters, chatter of the ECMO circuit, and intracardiac filling on TEE. Preparation for massive transfusion in this case included the placement of a RIC line, a rapid infusing device to provide a blood product reservoir external to the ECMO circuit, a cell saver salvage system, and preparation of sufficient blood products in anticipation of hemorrhage.

The venous reservoir in CPB also serves the important function of decreasing the risk that air entrained in the venous cannula will become an arterial air embolism. In addition, CPB also has the advantages of an in-circuit filter to reduce the risk of air emboli both during the case and during the transition back to the native circulation. The absence of a filter in VA-ECMO circuit placed the patient at unavoidable risk of unevacuated air in the PA after its closure. An open, right-sided procedure on ECMO was possible because of the body's physiologic filter: the lungs. Classic studies on venous air embolism in animal models found that air spillover to the arterial side only occurs at rates of air infusion exceeding 0.3 cc/kg/minute [4], placing the patient at low risk of paradoxical embolization from small volumes of residual intraoperative air in the PA. Establishing the absence of an intracardiac shunt (ie. PFO, atrial spetal defect (ASD)) or significant intrapulmonary shunt was particularly important in this case to assess the patient's risk for air embolization to the left heart. Patients with PE are at increased risk of right to left embolization due to the elevated right-sided pressures. Similar cases that do involve patients with an intracardiac shunt should have the intracardiac shunt closed prior to performing the surgical embolectomy to minimize the risk of systemic air embolization. Ongoing surveillance by TEE was critical during the case to monitor for both air entrainment, evidence of right-to-left shunting, and volume status.

This case represents the first report of pulmonary embolectomy performed with VA-ECMO support rather than CPB for a patient due to the presence of intracranial hemorrhage. The patient tolerated the surgical pulmonary embolectomy performed on VA-ECMO without procedure-related complications, and the ECMO support did not substantially complicate the technical performance of the procedure. Due to the lack of a blood reservoir, close attention must be paid to hemorrhage and signs of hypovolemia to ensure adequate resuscitation. This case suggests cardiopulmonary support on ECMO as a viable strategy for surgical embolectomy in patients with unstable PEs in whom thrombolysis or full systemic anticoagulation are contraindicated.

## Funding

None.

## Declaration of competing interest

None of the authors declare a conflict of interest.
